# Comparison of ^68^Ga-Prostate Specific Membrane Antigen (PSMA) Positron Emission Tomography Computed Tomography (PET-CT) and Whole-Body Magnetic Resonance Imaging (WB-MRI) with Diffusion Sequences (DWI) in the Staging of Advanced Prostate Cancer

**DOI:** 10.3390/cancers13215286

**Published:** 2021-10-21

**Authors:** Julien Van Damme, Bertrand Tombal, Laurence Collette, Sandy Van Nieuwenhove, Vassiliki Pasoglou, Thomas Gérard, François Jamar, Renaud Lhommel, Frédéric E. Lecouvet

**Affiliations:** 1 Department of Urology, Institut de Recherche Expérimentale et Clinique (IREC), Cliniques Universitaires Saint-Luc, Université Catholique de Louvain, B-1200 Brussels, Belgium; julien.vandamme@uclouvain.be (J.V.D.); bertrand.tombal@uclouvain.be (B.T.); 2 International Drug Development Institute (IDDI), B-1341 Louvain-la-Neuve, Belgium; laurence.collette@iddi.com; 3 Department of Radiology, Institut de Recherche Expérimentale et Clinique (IREC-IMAG), Cliniques Universitaires Saint-Luc, Université Catholique de Louvain, B-1200 Brussels, Belgium; sandy.vannieuwenhove@uclouvain.be (S.V.N.); vassiliki.pasoglou@uclouvain.be (V.P.); 4 Department of Nuclear Medicine, Institut de Recherche Expérimentale et Clinique (IREC-MIRO), Cliniques Universitaires Saint-Luc, Université Catholique de Louvain, B-1200 Brussels, Belgium; thomas.gerard@uclouvain.be (T.G.); francois.jamar@uclouvain.be (F.J.); renaud.lhommel@uclouvain.be (R.L.)

**Keywords:** PSMA, PET-CT, WB-MRI, prostate cancer, staging

## Abstract

**Simple Summary:**

Precise staging is key for the optimal management of advanced prostate cancer. PSMA PET-CT and WB-MRI outperform standard imaging technology for staging high-risk prostate cancer, but direct comparison between both modalities is lacking. The primary endpoint of our study was to compare the diagnostic accuracy of both techniques in the detection of lymph node, bone and visceral metastases against a best valuable comparator (BVC), defined as a consensus adjudication of all lesions on the basis of baseline and follow-up imaging, biological and clinical data and histopathologic confirmation when available. Knowing the diagnostic accuracy of both next generation imaging modalities might influence the diagnostic and therapeutic strategy in prostate cancer by tailoring therapy. However, the impact on treatment and patient outcome of an improved detection of metastases has not been determined yet.

**Abstract:**

Background: Prostate specific membrane antigen (PSMA) positron emission tomography computed tomography (PET-CT) and whole-body magnetic resonance imaging (WB-MRI) outperform standard imaging technology for the detection of metastasis in prostate cancer (PCa). There are few direct comparisons between both modalities. This paper compares the diagnostic accuracy of PSMA PET-CT and WB-MRI for the detection of metastasis in PCa. One hundred thirty-four patients with newly diagnosed PCa (*n* = 81) or biochemical recurrence after curative treatment (*n* = 53) with high-risk features prospectively underwent PSMA PET-CT and WB-MRI. The diagnostic accuracy of both techniques for lymph node, skeletal and visceral metastases was compared against a best valuable comparator (BVC). Overall, no significant difference was detected between PSMA PET-CT and WB-MRI to identify metastatic patients when considering lymph nodes, skeletal and visceral metastases together (AUC = 0.96 (0.92–0.99) vs. 0.90 (0.85–0.95); *p* = 0.09). PSMA PET-CT, however, outperformed WB-MRI in the subgroup of patients with newly diagnosed PCa for the detection of lymph node metastases (AUC = 0.96 (0.92–0.99) vs. 0.86 (0.79–0.92); *p* = 0.0096). In conclusion, PSMA PET-CT outperforms WB-MRI for the detection of nodal metastases in primary staging of PCa.

## 1. Introduction

Precise staging is key for the optimal management of advanced prostate cancer (PCa). It supports local treatment, and, increasingly, emerging therapies such as metastasis targeted therapies (MDT) [1]. Current treatment guidelines recognize thoraco-abdominal computed tomography (CT), abdominal magnetic resonance imaging and ^99m^Tc-bone scintigraphy (BS) as the cornerstone imaging techniques for metastatic assessment in PCa. There is compelling evidence that emerging imaging technologies such as ^68^Ga-PSMA-11 positron emission tomography CT (PSMA PET-CT) and whole-body magnetic resonance imaging (WB-MRI) with diffusion weighted imaging (DWI) outperform bone scintigraphy and thoraco-abdomino-pelvic CT for the detection of PCa metastases [2,3,4,5,6].

Despite this evidence, international guidelines still do not recommend the use of these new imaging techniques, apart for PSMA PET-CT which is now acknowledged as a standard of care in patients presenting with a PSA recurrence (BCR) after local treatment [7,8,9]. One of the main reasons, is the limited evidence that this increased diagnostic accuracy positively impacts the PCa care pathway and results in improved oncological outcome. Hence, detection of metastases may reclassify patients with newly diagnosed (ND) PCa from high-risk localized to metastatic with the consequence of omitting local treatment or consolidating systemic treatment with a last generation androgen receptor targeted agent (ARTA). In patients with biochemical recurrence after local treatment, more rapid detection of low volume metastatic deposit has boosted the emergence of metastatic directed ablative strategies in absence of clinical equipoise, since this approach is only supported by small phase II trials [10].

Several whole-body imaging techniques compete to replace BS and CT as standard of care imaging, but, unfortunately, there is limited data available to compare their diagnostic accuracy. Hence, this study compares PSMA PET-CT and WB-MRI for the detection of lymph node, bone and visceral metastases in PCa patients.

## 2. Materials and Methods

### 2.1. Study Design

This retrospective study of data prospectively collected in a single institution was conducted between 12/2016 and 12/2019 in 155 consecutive patients with newly diagnosed PCa at high risk for metastases (patients were included only if 2 or the 3 high-risk criteria were present: PSA > 20 ng/mL, Gleason score ≥8 or ≥cT3) or PSA recurrence after local treatment (defined as a PSA level >0.2 ng/mL after radical prostatectomy or >2 ng/mL after radiation therapy with a PSA doubling time <12 months). The trial was approved by the local ethical committee. After consenting, patients underwent both ^68^Ga-PSMA PET-CT and WB-MRI including DWI sequences within 1 month.

“Standard” imaging modalities (CT and BS) were not systematically performed, as both PSMA PET-CT and WB-MRI are available in our center and as the diagnostic superiority of these two techniques has been repeatedly established [2,3,4,5,6]. Patients were treated at the referring physician’s discretion based on international recommendations and were followed up for at least 12 months.

### 2.2. Imaging Methods

Imaging protocols were predefined, both for PSMA PET-CT including PET-CT acquisition parameters, quality control and specifications for the radiopharmaceutical production, and for WB-MRI examinations that were designed after the METastasis Reporting and Data System (METRADS) guidelines for PCa [11].

#### 2.2.1. PSMA-PET/CT Acquisition and Readings

^68^Ga-PSMA-11 radio-pharmaceutical was synthesized onsite as previously described in good medical practice environment using a Scintomics GRP4V synthesizer (PSMA-HBED-11 labelling kits provided by ABX, Germany; ^68^Ge/^68^Ga Galli Ad generator, IRE Elite, Fleurus, Belgium). All ^68^Ga-PSMA PET/CT examinations were performed in clinical routine conditions after intravenous injection of a target activity of 110 MBq (mean, 123 ± 33 MBq, range 73–207 MBq) [12]. PET/CT images were acquired at least 60 min post injection (mean 79 ± 17 min, ranging from 59 to 137 min p.i), either on Gemini-TF 64 slices or Vereos digital PET/CT (Philips Healthcare, Best, The Netherlands). Low dose CT was acquired first for attenuation correction, then followed, when no contraindication was present, by a diagnostic thoraco-abdomino-pelvic CT with iodinated contrast injection in portal phase (Iobitridol, Xenetix 350 mg/mL, Guerbet, Roissy, France) and dose-reduction protocol (iterative reconstruction/dose modulation). PET/CT were acquired from the vertex to mid-thighs with arms above the head in 3D mode and reconstructed following standard manufacturer parameters (isometrics voxels of 4 mm for the Gemini-TF and 4–2 mm for the Vereos) after usual attenuation, scatter and time-of-flight corrections. Furosemide was not applied routinely. Perfusion of 500 mL 0.9% saline before injection was used to correctly hydrate the patient during the incorporation time. No dedicated fasting instructions were required for the PSMA PET/CT.

Two board certified nuclear medicine physicians performed all readings in consensus in a randomized order, blinded to clinical data and other imaging results. All examinations were reviewed using Osirix MD software (Pixmeo SARL, Bernex, Switzerland).

Eight anatomical regions for bones (skull, thoracic cage, cervical spine, thoracic spine, lumbar spine, pelvis, humeri, femurs) and seven regions for nodes (inguinal, internal and external iliac together, common iliac, lumbo-aortic, thoracic, axillary, cervical regions) were considered for the metastatic assessment.

Regarding bones, in each region, the bone marrow was considered as being either normal or presenting focal or diffuse tumoral involvement. Regarding lymph nodes, moderate (at least superior to the blood pool) to intense focal nodal uptake was considered for positivity without any consideration of the node size, especially within the pelvic and retroperitoneum area which are at higher risk of lymph node involvement in both ND and BCR stages. Visceral metastases were recorded by organ (mainly lung, liver and peritoneal cavity/carcinomatosis). The number and the presence of metastases were recorded in each region and the metastatic status was summarized in a per-organ approach (bones, nodes, visceral lesions present or not) and in a per-patient approach (metastatic or not).

#### 2.2.2. WB-MRI Acquisition and Readings

WB-MRI examinations were performed on a 3.0-T MRI magnet (Ingenia, Philips Medical Systems, Best, The Netherlands). Five stacks of coronal 3D fast spin echo (FSE) T1, STIR, and axial DWI (three b-values: 0, 150 and 1000 s/mm^2^) images were obtained, covering the body from the vertex to mid-thighs, as recommended in the METRADS guidelines, and using previously published imaging parameters [11,13,14]. Total image acquisition time was less than 45 min.

Two board-certified musculoskeletal radiologists with more than 10 years’ experience in WB-MRI reading performed all readings in consensus in a randomized order, blinded to clinical data and other imaging results. All images were read on PACS workstations (Carestream Vue; Carestream Health, Rochester, NY, USA), using the multiplanar reformation, link, scroll and zoom tools for side-by-side reading of anatomical and DWI sequences.

In each region, the bone marrow was considered as being either normal or presenting either focal or diffuse metastatic involvement based on widely accepted categorization criteria [15,16].

Lymph nodes were considered abnormal on WB-MRI when their short-axis diameter was larger than 10 mm. Perivisceral (perivesical, perirectal, etc.) nodes were defined as abnormal when their short-axis diameter was larger than 8 mm. Lymph nodes were also considered abnormal when their contour was irregular or when there was loss of either the normal kidney shape, of the fatty hilum, or both. This widely accepted categorization has been repeatedly described [13,17,18,19,20].

The same per-organ and per-patient summary was used as for PSMA-PET/CT. For both PSMA PET/CT and WB-MRI, additional relevant information provided by the whole-body examinations about the local PCa status or incidental findings (e.g., kidney, lung, brain tumors, etc.) were not considered in this study, being beyond its scope.

### 2.3. Reference Standard or “Best Valuable Comparator”

In the absence of a systematic histologic gold standard, a “best valuable comparator” (BVC) was used to adjudicate the presence of metastases at the region, organ and patient levels, in line with previous diagnostic trials [21,22,23]. The BVC included results from all available baseline and follow-up imaging modalities (modern, i.e., PSMA PET-CT and WB-MRI, and standard, i.e., BS and CT when available), clinical follow-up data (PSA kinetics, skeletal events), histopathological proofs when available after biopsy of lesions (bone, lymph nodes or visceral) or surgical treatment (salvage lymph node dissection). A multidisciplinary panel including one nuclear medicine physician and one radiologist distinct from those who had performed the prospective readings, one urologist, one medical oncologist and one pathologist determined the benign or malignant nature of each lesion reported from each modality based on this BVC.

### 2.4. Endpoints

The primary endpoint was to compare the diagnostic performance of WB-MRI and PSMA PET-CT for detecting metastases at the patient level using BVC as reference standard. The secondary endpoints were to compare the diagnostic performance of WB-MRI and PSMA PET-CT for detecting lymph nodes, bone and visceral metastases separately, and to measure the inter-reader agreement of WB-MRI and of PSMA PET-CT at the lymph node/bone/visceral and patient levels. The analyses were also performed in the ND and BCR subpopulations separately.

### 2.5. Statistical Analysis

The diagnostic accuracy of each modality is described by its sensitivity, specificity, positive predictive value, negative predictive value, with their 95% exact confidence interval and summarized by the area under the receiver operating characteristic (ROC) curves (AUC = (sensitivity + specificity)/2). These values of imaging modalities to detect metastases are expressed at the patient level and by organ (bones, lymph nodes, visceral metastases). The AUCs were compared by the Wald test from a logistic regression model.

False positive and negative rates were also reported for each modality at each level.

Analyses were performed for the whole population and separately for the ND PCa population and for the BCR population. All tests and confidence intervals (CI) are 2-sided with α = 0.05. The statistical analysis software, version 9.4 (SAS, Bethesda, MD, USA) was used for these analyses.

Inter-rater agreement was measured using prevalence-adjusted bias-adjusted kappa coefficients [24]. The study is exploratory and the sample size was not prospectively planned. No multiplicity adjustment was applied.

## 3. Results

**Figure 1:**
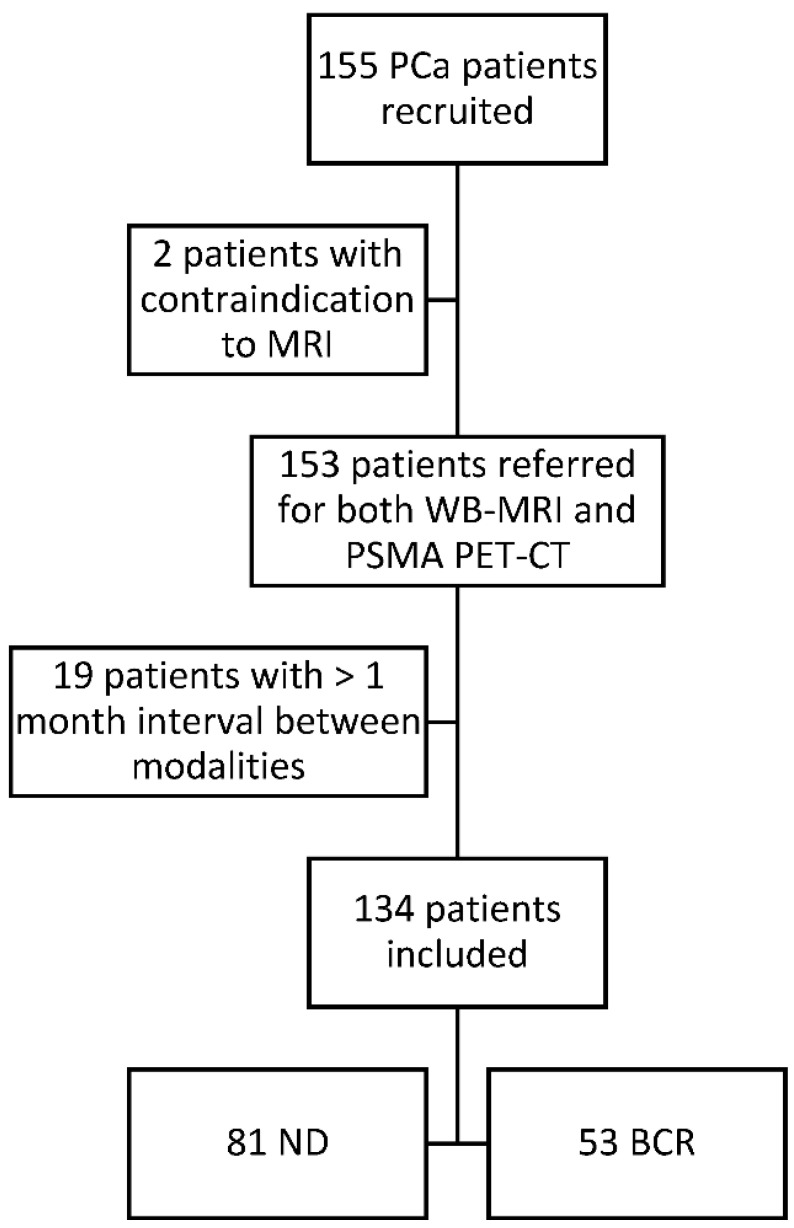
Study flow chart. All patients were diagnosed with biopsy-proven prostate cancer and all patients underwent a WB-MRI and a PSMA PET-CT at the University Clinic Saint-Luc Brussels from August 2016 to November 2019. PCa: prostate cancer; WB-MRI: whole body magnetic resonance imaging; PSMA PET-CT: prostate specific membrane antigen positron emission tomography; ND: newly diagnosed; BCR: biochemical recurrence.

Of the 155 clinically eligible patients, 2 were excluded because of contraindication to MRI (pacemaker) and 19 for an interval between the imaging procedures exceeding 1 month. The median time interval between examinations was 8 days (IQR: 15 days; SD: 20.05 days). The study flowchart is presented in Figure 1. The baseline clinical characteristics of the 134 patients included in the final analysis are detailed in Table 1. Eighty-one patients were newly diagnosed high-risk PCa and 53 were presenting with a biochemical recurrence after surgery or radiotherapy.

### 3.1. Primary Endpoint

Based on the BVC, 66/134 patients (49%) were adjudicated metastatic and 68/134 (51%) non-metastatic (Table 2). Thirty-three patients (25%) had at least one bone metastasis, 51 (38%) had pathologic lymph nodes and 8 (5%) had visceral metastases. PSMA PET-CT detected metastases in 65 of the 66 metastatic patients considered metastatic by the BVC (true positive) and in 5 of the 68 non-metastatic patients (false positive). WB-MRI detected metastases in 56 of the 66 considered metastatic patients by BVC (true positive), and in 3 of the 68 the non-metastatic patients (false positive). Table 3 details the discordant cases. The AUC for identifying metastatic patients was 0.96 (95% CI, 0.92–0.99) and 0.90 (0.85–0.95) for PSMA PET-CT and WB-MRI, respectively (*p* = 0.09). The inter-technique agreement was relatively good but far from perfect (adjusted kappa = 0.72; 0.60–0.83).

### 3.2. Secondary Endpoints

At the organ level, PSMA PET-CT and WB-MRI detected 180 and 177 bone metastases, 217 and 141 pathologic lymph nodes and 9 and 6 visceral metastases, respectively. The AUC for identifying bone metastases was 0.95 (0.92–0.99) and 0.98 (0.95–1.00) for PSMA PET-CT and WB-MRI, respectively (*p* = 0.32). WB-MRI detected all bone metastases resulting in 100% sensitivity, whereas PSMA PET-CT missed one bone lesion. The AUC for identifying lymph node metastases was 0.96 (0.92–0.99) and 0.86 (0.79–0.92) for PSMA PET-CT and WB-MRI, respectively (*p* = 0.0096).

### 3.3. In the Subgroup of Patients with Newly Diagnosed PCa

**Figure 2:**
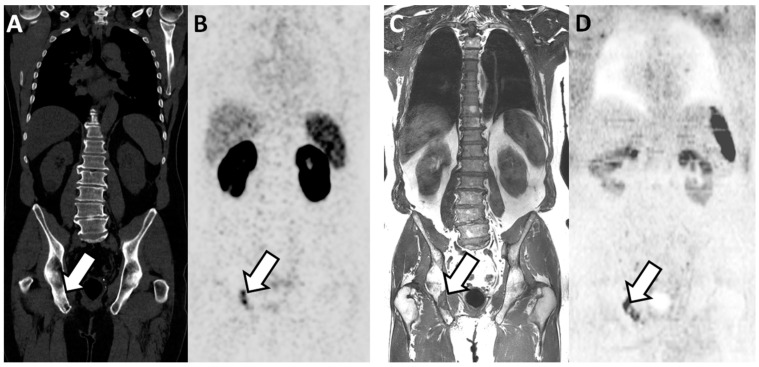
Concordant findings between PSMA PET/CT and WB-MRI/DWI: true positive observation of bone metastasis by WB-MRI/DWI in 65-year-old patient with ND prostate cancer at high risk for metastasis. (**A**,**B**) PSMA PET/CT: reformatted coronal CT (**A**) and PET (**B**) images show sclerotic lesion within the right ilio-ischiatic ramus with evident tracer uptake (arrows in (**A**,**B**)). (**C**,**D**) WB-MRI/DWI images: corresponding reformatted high resolution T1 (**C**) and diffusion-weighted (**D**) MRI images show bone lesion of low signal intensity on T1 and high signal intensity on MR images (inverted grey scale in the figure) within the right ilio-ischiatic ramus. The best valuable comparator confirmed the metastatic nature of the lesion.

PSMA PET-CT significantly outperformed WB-MRI (Table 4). At the patient level, the AUC was 0.96 (0.92–1.00) and 0.87 (0.79–0.95) for PSMA PET-CT and for WB-MRI, respectively (*p* = 0.045). The inter-technique agreement was good (adjusted kappa = 0.70; 0.55–0.86). The AUC for pathological lymph node detection was 0.98 (0.96–1.00) and 0.83 (0.74–0.92) for PSMA PET-CT and WB-MRI, respectively (*p* = 0.002). The AUC for bone metastases detection was 0.94 (0.87–1.00) and 0.99 (0.96–1.00) for PSMA-PET and WB-MRI, respectively (*p* = 0.24) (Figure 2). At the bone level, WB-MRI detected all lesions, whereas PSMA PET-CT missed one.

### 3.4. In the Subgroup of Patients with Biochemical Recurrence after Local Treatment

**Figure 3:**
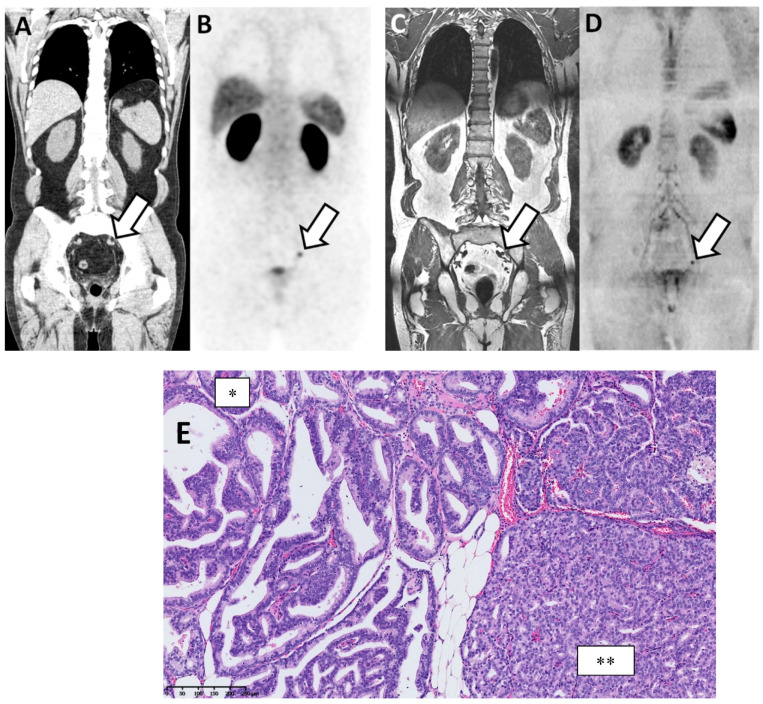
Discordant findings between PSMA PET/CT and WB-MRI/DWI: false negative observation of metastatic lymph node by WB-MRI/DWI in 62-year-old patient with prostate cancer at BCR (rapidly rising PSA up to 3.5 ng/mL after radical prostatectomy and pelvic irradiation). (**A**,**B**) PSMA PET/CT: reformatted coronal CT (**A**) and PET (**B**) images show unique 10 mm radiotracer-positive (SUVmax 12) left iliac lymph node considered as metastatic on the PET image (arrows in (**A**,**B**)). (**C**,**D**) WB-MRI/DWI images: corresponding reformatted high resolution T1 (**C**) and diffusion-weighted (**D**) MRI images show single left lymph node (arrows in (**C**,**D**)) that was not considered as metastatic based on its size (small diameter < 10 mm). (**E**) Hematoxylin and eosin stained section of resected node after robotic-assisted salvage lymphadenectomy shows the presence of a PCa metastasis with a mixed intra-ductal (*) and cribriform (**) morphology (original magnification ×300; scale bar in lower left corner).

PSMA PET-CT had higher sensitivity and specificity without reaching statistical significance (Table 5). At the patient level, the AUC for identifying metastatic patients was 0.96 (0.90–1.00) and 0.89 (0.79–0.98) for PSMA PET-CT and WB-MRI, respectively (*p* = 0.25). WB-MRI missed one additional patient (false negative) compared to the PSMA PET-CT. The inter-technique agreement was good (adjusted kappa = 0.74; 0.55–0.92). At the lymph node level, the AUC was 0.94 (0.87–1.00) and 0.88 (0.79–0.97) for PSMA PET-CT and WB-MRI, respectively (*p* = 0.34) (Figure 3). At the bone level, both modalities performed equally well with an AUC of 0.96 (0.90–100).

## 4. Discussion

New imaging techniques providing high resolution whole-body anatomical coverage and functional information on tissues are clearly reshaping the PCa treatment landscape. It is only a matter of time before they replace BS and CT, depending on local accessibility and development of expertise to address the different clinical needs and referring physician expectations. Besides local availability and costs, physicians will have to choose amongst different techniques based on their diagnostic effectiveness, which requires objective comparison studies.

The primary endpoint of our study was to compare the diagnostic accuracy of PSMA PET-CT and WB-MRI to detect bone, lymph node and visceral metastases in a broad group of patients with newly diagnosed and recurrent PCa, thus reflecting current indications. After adjudication with a best valuable comparator, metastases were found in 38% of the ND patients and 66% of the BCR patients, in line with previous reports [5,21,22,25].

In the present study, BVC adjudicated 5 false positive and 1 false negative results with PSMA PET-CT and 3 false positive and 10 false negative results with WB-MRI. There was no significant difference in AUCs.

For the detection of lymph node metastases, PSMA PET-CT clearly outperformed WB-MRI, resulting from a higher sensitivity of PSMA PET-CT to detect small metastatic lymph nodes. These findings are in line with the results from the systematic review and meta-analysis of Perera et al., highlighting the high sensitivity and specificity of PSMA PET-CT to detect small lymph nodes at low PSA levels [5]. WB-MRI has a lower sensitivity and specificity for lymph node detection because of its inability to detect tumoral infiltration in normal size lymph nodes [18]. Noteworthily, in absence of systematic pathological verification, the BVC used in this study cannot rule-out all false positive findings of imaging. In a study by Montorsi et al., 16 patients with BCR after local treatment underwent salvage lymph dissection based on a positive PSMA PET-CT, and positive lymph nodes were identified at pathologic analysis in only 68% of the patients, thus 32% being false positive [26]. A study by Herlemann et al. comparing PSMA PET-CT and histologic findings after lymph node dissection found lower false positive rates for lymph nodes detected by PSMA-PET (less than 8% in the region-based analysis, 12% in the patient-based analysis) [27]. Another report, comparing PSMA PET/CT to pathological findings after template pelvic lymph node dissection in 130 patients, underlined the excellent specificity (98.9%) and accuracy (88.5%) of PSMA PET/CT for metastatic lymph node detection, but also pointed out its lower effectiveness to detect small truly positive lymph nodes (sensitivity of 65.9%, compared to pathological findings) [28].

In contrast to lymph nodes, PSMA PET/CT and WB-MRI performed equally for the detection of bone metastases, with PSMA PET-CT missing only one bone metastasis. Our study thus confirms the high diagnostic performance of WB-MRI for bone lesions detection that was primarily demonstrated in comparison with conventional techniques [22,29,30,31]. This study, however, suggests that PSMA PET-CT reaches similar diagnostic accuracy for bone metastasis detection, in line with published comparisons between MRI and PET/CT using either choline or PSMA ligands [32,33].

In contrast to conventional imaging, that indirectly identifies the presence of bone metastasis by highlighting the bone sclerosis or osteolysis induced by metastatic cells, ^68^Ga-PSMA-PET-CT and WB-MRI directly identify tumoral cell deposits within the skeleton. PSMA PET-CT targets membrane antigens on the surface of metastatic cells. Although the uptake of ^68^Ga-PSMA-PET is higher in osteolytic metastases compared to osteoblastic lesions due to a lower tumor cell content, abundant literature shows that both lytic and sclerotic bone lesions can be accurately imaged by PSMA PET/CT, at least as long as the PSMA expression is preserved [34,35]. MRI detects the replacement of the normal bone marrow content by neoplastic cells which leads to alteration of the signal intensity on the morphologic sequences and alteration of water diffusivity on molecular DWI sequences.

Interestingly, a high rate of false positive bone lesions (12/79) has been reported in the PROSTAGE PSMA PET-CT study [21]. In the current study, PSMA PET-CT and WB-MRI only had 5 and 6 false positive bone lesions, respectively. The false positive PSMA PET-CT and WB-MRI were both due to degenerative changes, benign fractures or benign bone lesions (observed in different patients for PSMA PET-CT and WB-MRI). This is in agreement with the literature, which reports that fractures, degenerative joint disease, hemangiomas, Paget’s disease or fibrous dysplasia may be challenging for both PSMA PET-CT and WB-MRI [36]. Regarding this point, training of the different readers is crucial to identify the frequent pitfalls of both techniques.

PSMA PET-CT mainly outperformed WB-MRI in the subgroup of ND PCa for the detection of nodal metastases, confirming the results of previous randomized controlled trials. In the PROSTAGE study, 80 ND patients with high-risk features were assigned to bone scintigraphy, computed tomography, SPECT-CT, WB-MRI and ^18^F-PSMA PET-CT for primary staging. PSMA PET-CT outperformed all other imaging modalities for the detection of primary distant metastases. At patient level, the AUC (optimistic analysis) was 0.89 (0.80–0.97), 0.88 (0.80–0.97), 0.85 (0.76–0.94) and 0.81 (0.71–0.91) for PSMA PET-CT, WB-MRI, SPECT-CT and CT, respectively [21]. In the ProPSMA study (*n* = 302), ^68^Ga-PSMA PET-CT had a significantly higher accuracy than conventional imaging (92% (88–95) vs. 65% (60–69); *p* < 0.0001) [25]. A recent prospective trial by Malaspina compared the performance of ^18^F-PSMA-1007 PET/CT, WB-MRI with DWI and CT for the primary pelvic nodal staging of PCa using robot-assisted laparoscopic prostatectomy and pelvic lymph node dissection as gold standard. Thirty-nine percent of patients had pelvic lymph node metastases. PSMA PET/CT, WB-MRI and CT detected 87%, 45% and 26% of the lesions, respectively. At the patient level, the inter-reader agreement and concordance with histopathology findings were higher for PSMA PET-CT compared to those of WB-MRI and CT (kappa values of respectively 0.89 vs. 0.47 and 0.69, respectively [37]. Noteworthy, these authors also demonstrated that 74% of the metastatic lymph nodes were smaller than the anatomical cut-off size of 8 mm, confirming our previous report that 70% of PSMA PET-CT positive lymph nodes were presenting a size below 8 mm, with a smallest positive lesion size of 1.7 mm [38].

In contrast, our results show that PET-PSMA and WB-MRI performed equally well at the patient level in the subgroup of patients with BCR after local therapy. Although it did not reach statistical significance, our study suggests that WB-MRI may lead to an underestimation of the global metastatic load, in case of small lymph node involvement.

Our study does not include a cost-effectiveness analysis. The high cost of new generation imaging is increasingly recognized as an important issue in the healthcare system, although it fluctuates between countries and continents. The costs of WB-MRI or PSMA PET-CT, offering a one-step and accurate diagnostic approach, should be put in balance against the use of multiple techniques and against the cost of futile or harmful therapeutic strategies based on inaccurate information about the real metastatic status. An additional advantage of WB-MRI over PET/CT is that MRI does not use ionizing radiation.

## 5. Conclusions

In this study, PSMA PET-CT outperformed WB-MRI to detect metastases, mainly due to a higher accuracy for metastatic lymph node detection in the primary staging of newly diagnosed patients. Although PSMA PET-CT has become the most widely accepted imaging technique, WB-MRI can be an alternative.

## Figures and Tables

**Table 1:** Patient demographics, disease characteristics and primary treatment.

Variable	Newly Diagnosed (*n* = 81)	Biochemical Recurrence (*n* = 53)
Age, year		
Median (IQR)	67 (62–72)	67 (62–73)
PSA, ng/mL		
Median (IQR)	12.29 (7.93–29)	6.85 (1.44–13.67)
Clinical Stage, cT		
cT1	40	27
cT2	25	17
cT3	11	7
cT4	5	2
ISUP grade group		
1	1	7
2	5	15
3	7	13
4	30	11
5	38	7
D’AMICO risk group classification		
Low	1	8
Intermediate	5	27
High	75	18
Primary treatment		
WAWA	1	0
RP	16	17
EBRT	51	31
Brachy	1	5
ADT + ARTA	7	0
ADT + chemo	5	0

PSA: prostate specific antigen; ISUP: International Society of Urological Pathology; WAWA: watchful waiting; RP: radical prostatectomy; EBRT: external beam radiotherapy; Brachy: brachytherapy; ADT: androgen deprivation therapy; ARTA: antiandrogen receptor targeted agent; Chemo: chemotherapy.

**Table 2:** Comparison of the diagnostic effectiveness of PSMA PET-CT and WB-MRI in 134 patients with prostate cancer (at the organ and patient levels).

Variable	Patient Level		Bone Level		Lymph Node Level		Visceral Level	
Imaging Modality	PSMA PET-CT	WB-MRI	PSMA PET-CT	WB-MRI	PSMA PET-CT	WB-MRI	PSMA PET-CT	WB-MRI
Sensitivity	0.98 (0.96–1.00)	0.85 (0.76–0.94)	0.97 (0.91–1.00)	1.00 (1.00–1.00)	0.94 (0.88–1.00)	0.73 (0.60–0.85)	1.00 (1.00–1.00)	0.75 (0.45–1.00)
Specificity	0.93 (0.86–0.99)	0.96 (0.90–1.00)	0.94 (0.89–0.99)	0.95 (0.91–0.99)	0.98 (0.94–1.00)	0.99 (0.96–1.00)	0.99 (0.98–1.00)	1.00 (1.00–1.00)
Positive Predictive Value	0.92 (0.87–0.99)	0.95 (0.89–1.00)	0.84 (0.73–0.96)	0.87 (0.76–0.98)	0.96 (0.91–1.00)	0.97 (0.92–1.00)	0.89 (0.68–1.00)	1.00 (1.00–1.00)
Negative Predictive Value	0.98 (0.95–1.00)	0.87 (0.79–0.94)	0.99 (0.97–1.00)	1.00 (1.00–1.00)	0.96 (0.92–1.00)	0.85 (0.78–0.92)	1.00 (1.00–1.00)	0.98 (0.96–1.00)
False Positive	5	3	6	5	2	1	1	0
False Negative	1	10	1	0	3	14	0	2
Diagnostic Accuracy	0.96 (0.92–0.99)	0.90 (0.85–0.95)	0.95 (0.92–0.99)	0.98 (0.95–1.00)	0.96 (0.92–0.99)	0.86 (0.79–0.92)	1.00 (0.99–1.00)	0.88 (0.71–1.00)
*p* value comparing AUC *	0.09		0.32		0.0096		0.14	
Cohen’s Kappa PET-CT vs. WB-MRI **	0.72 (0.60–0.83)		0.88 (0.80–0.96)		0.70 (0.58–0.82)		0.96 (0.90–1.00)	

WB-MRI: whole body magnetic resonance imaging; PSMA PET-CT: prostate specific membrane antigen positron emission tomography; AUC: area under the curve; * all *p* values of <0.05 were considered statistically significant; ** The inter-technique agreement was defined using adjusted Cohen’s kappa; all values between parentheses are the 95% confidence interval.

**Table 3:** Causes of false positive and negative observations in the patient-based analysis of the whole patient population, as adjudicated after the analysis of the files by the time of BVC.

Variable	PSMA PET-CT	WB-MRI
**Bone**		
False Positive	*N* = 6	*N* = 5
	Degenerative changes (*n* = 2) Benign fractures (*n* = 2) Benign bone lesion (*n* = 1)	Benign fractures (*n* = 2) Degenerative changes (*n* = 2) Hemangioma (*n* = 1)
False Negative	*N* = 1	*N* = 0
	Lack of PSMA expression	
**Lymph Node**		
False Positive	*N* = 2	*N* = 1
	Subtle uptake in benign LN (*n* = 2)	Inflammatory adenopathy
False Negative	*N* = 3	*N* = 14
	Small nodes without uptake shown metastatic at follow-up (*n* = 2) Subtle uptake without CT abnormality (*n* = 1)	Small size of metastatic nodes (<10 mm) (*n* = 12) Missed mediastinal LN (*n* = 1) Missed inguinal LN (*n* = 1)
**Viscera**		
False Positive	*N* = 1	*N* = 0
	Benign liver lesion	
False Negative	*N* = 0	*N* = 2
		Lung metastases (*n* = 2)

PSMA: prostate specific membrane antigen; LN: lymph node.

**Table 4:** Comparison of the diagnostic effectiveness of WB-MRI and PSMA PET-CT in 81 newly diagnosed prostate cancer patients.

Variable	Patient Level		Bone Level		Lymph Node Level		Visceral Level	
Imaging Modality	PSMA PET-CT	WB-MRI	PSMA PET-CT	WB-MRI	PSMA PET-CT	WB-MRI	PSMA PET-CT	WB-MRI
Sensitivity	1.00 (1.00–1.00)	0.74 (0.59–0.90)	0.93 (0.79–1.00)	1.00 (1.00–1.00)	1.00 (1.00–1.00)	0.67 (0.49–0.84)	1.00 (1.00–1.00)	1.00 (1.00–1.00)
Specificity	0.92 (0.84–0.99)	1.00 (1.00–1.00)	0.95 (0.90–1.00)	0.97 (0.93–1.00)	0.96 (0.91–1.00)	1.00 (1.00–1.00)	0.99 (0.96–1.00)	1.00 (1.00–1.00)
Positive Predictive Value	0.88 (0.78–0.99)	1.00 (1.00–1.00)	0.81 (0.62–1.00)	0.87 (0.71–1.00)	0.93 (0.84–1.00)	1.00 (1.00–1.00)	0.75 (0.33–1.00)	1.00 (1.00–1.00)
Negative Predictive Value	1.00 (1.00–1.00)	0.86 (0.77–0.95)	0.98 (0.95–1.00)	1.00 (1.00–1.00)	1.00 (1.00–1.00)	0.86 (0.77–0.94)	1.00 (1.00–1.00)	1.00 (1.00–1.00)
False Positive	4	0	3	2	2	0	1	0
False Negative	0	8	1	0	0	9	0	0
Diagnostic Accuracy	0.96 (0.92–1.00)	0.87 (0.79–0.95)	0.94 (0.87–1.00)	0.99 (0.96–1.00)	0.98 (0.96–1.00)	0.83 (0.74–0.92)	0.99 (0.99–1.00)	1.00 (1.00–1.00)
*p* value comparing AUC *	0.045		0.24		0.002		0.32	
Cohen’s Kappa PET-CT vs. WB-MRI **	0.70 (0.55–0.86)		0.95 (0.88–1.00)		0.73 (0.58–0.88)		0.98 (0.93–1.00)	

WB-MRI: whole body magnetic resonance imaging; PSMA PET-CT: prostate specific membrane antigen positron emission tomography; AUC: area under the curve; * all *p* values of <0.05 were considered statistically significant; ** The inter-technique agreement was defined using adjusted Cohen’s kappa; all values between parentheses are the 95% confidence interval.

**Table 5:** Comparison of the diagnostic effectiveness of WB-MRI and PSMA PET-CT in 53 PCa patients at biochemical recurrence.

Variable	Patient Level		Bone Level		Lymph Node Level		Visceral Level	
Imaging Modality	PSMA PET-CT	WB-MRI	PSMA PET-CT	WB-MRI	PSMA PET-CT	WB-MRI	PSMA PET-CT	WB-MRI
Sensitivity	0.97 (0.92–1.00)	0.94 (0.87–1.00)	1.00 (1.00–1.00)	1.00 (1.00–1.00)	0.87 (0.74–1.00)	0.79 (0.63–0.95)	1.00 (1.00–1.00)	0.60 (0.17–1.00)
Specificity	0.94 (0.84–1.00)	0.83 (0.66–1.00)	0.91 (0.82–1.00)	0.91 (0.82–1.00)	1.00 (1.00–1.00)	0.97 (0.90–1.00)	1.00 (1.00–1.00)	1.00 (1.00–1.00)
Positive Predictive Value	0.97 (0.92–1.00)	0.92 (0.83–1.00)	0.86 (0.72–1.00)	0.86 (0.72–1.00)	1.00 (1.00–1.00)	0.95 (0.85–1.00)	1.00 (1.00–1.00)	1.00 (1.00–1.00)
Negative Predictive Value	0.94 (0.84–1.00)	0.88 (0.73–1.00)	1.00 (1.00–1.00)	1.00 (1.00–1.00)	0.90 (0.80–1.00)	0.85 (0.73–0.97)	1.00 (1.00–1.00)	0.96 (0.91–1.00)
False Positive	1	3	3	3	0	1	0	0
False Negative	1	2	0	0	3	5	0	2
Diagnostic Accuracy	0.96 (0.90–1.00)	0.89 (0.79–0.98)	0.96 (0.90–1.00)	0.96 (0.90–1.00)	0.94 (0.87–1.00)	0.88 (0.79–0.97)	1.00 (1.00–1.00)	0.80 (0.56–1.00)
*p* value comparing AUC *	0.25		1		0.34		0.10	
Cohen’s Kappa PET-CT vs. WB-MRI **	0.74 (0.55–0.92)		0.77 (0.60–0.94)		0.66 (0.46–0.86)		0.92 (0.82–1.00)	

WB-MRI: whole body magnetic resonance imaging; PSMA PET-CT: prostate specific membrane antigen positron emission tomography; AUC: area under the curve; * all *p* values of <0.05 were considered statistically significant; ** The inter-technique agreement was defined using adjusted Cohen’s kappa; all values between parentheses are the 95% confidence interval.

## Data Availability

The data presented in this study are available on request from the corresponding author. The data are not publicly available due to ethical and privacy reasons.

## Abbreviations

ARTA	androgen receptor targeted agents
AUC	area under the curve
BCR	biochemical recurrence
BS	Bone scintigraphy
BVC	best valuable comparator
CT	computed tomography
TAP	thoraco-abdomino-pelvic
MDT	metastasis targeted therapies
ND	newly diagnosed
NGI	next generation imaging
PCa	prostate cancer
PSMA PET-CT	prostate specific membrane antigen positron emission tomography computed tomography
PSA	prostate specific antigen
ISUP	international society of uropathological pathology
WB-MRI	whole body magnetic resonance imaging
